# The Effects of Blue Light and Supplemental Far-Red on an In Vitro Multiple Harvest System for the Production of *Cannabis sativa*

**DOI:** 10.3390/plants14060966

**Published:** 2025-03-19

**Authors:** Molly McKay, James E. Faust, Matthew Taylor, Jeffrey Adelberg

**Affiliations:** 1 Plant and Environmental Sciences Department, 171 Poole Agricultural Center, Clemson University, Clemson, SC 29630, USA; jfaust@clemson.edu; 2 Curio Wellness, P.O. Box 5470, Towson, Baltimore, MD 21285, USA; matt.taylor@curiowellness.com

**Keywords:** far-red light, blue light, cannabis, micropropagation, LED, hedging

## Abstract

Blue and supplemental far-red light were observed to affect in vitro shoot growth with *Cannabis sativa* (‘BaOx’ and ‘Cherry 1’) in RV750 vessels. A modified “hedging” and fed-batch system for multiple harvests using Oasis^®^ foam and 120 mL DKW medium was used. Fifteen nodal and/or apical tips were planted and placed into PAR light treatments providing various red to blue ratios (polychromatic white 9:1 RB and dichromatic 2–15: 1 RB, with and without 5% far-red light). Treatments had similar light intensities (190–240 µmol · m^−2^ · s^−1^ PPFD) for a 16 h photoperiod. Shoot tips were harvested in vitro on five successive two-week cycles, with 15 mL of DKW media supplemented to each vessel following harvest. Shoot numbers, length, and fresh and dry mass were recorded at each cycle harvest. Five randomly selected shoot tips per vessel were rooted ex vitro on greenhouse mist bench for 16 days. Over multiple cycles, 5% far-red increased shoot numbers and length in both genotypes tested, regardless of polychromatic or dichromatic source. Shoots harvested per vessel increased from 15 to 28 in three cycles (6 weeks), but increased from 15 to 18 without far-red treatment. Shoot length in far-red-treated plants increased from 19 to 25 mm during cycles 1–3. Plants without far-red treatment were approximately 15 mm during the first three cycles. By cycle 5, both far-red- and non-far-red-treated plants decreased to 10 mm. Dry mass was greatest in cycle 1 for both genotypes (‘Cherry 1’ was 6 mg and ‘BaOx’ was 7 mg) under the highest amount of blue light, but 2 mg under the lowest amount of blue light. Dry mass decreased by 50% in cycle 3, to 4 mg, where it remained for the duration of the experiment. Sixty eight percent of shoots rooted ex vitro on the mist bench, regardless of any prior in vitro treatment.

## 1. Introduction

Light is an environmental factor that affects plant morphology. While sunlit conditions vary with season (intensity, quality), electrically lit environments offer more controlled environmental and light conditions, with space efficiency increased by vertical stacking. Light-emitting diodes (LEDs) are increasingly chosen as the light sources for tissue culture laboratories to control spectral composition, with the potential to increase yield [1]. An effective and efficient micropropagation protocol depends on the proliferation of shoots and stability in numbers of explants [2]. LED light sources can be used monochromatically, dichromatically in ratios such as R:B, or poly-chromatically in lamps perceived as white light. Originally, white LEDs were used in the general lighting applications of homes, offices, and streetlamps. These lights were not specialized for plant growth or spectrally balanced for that purpose; however, due to their lower cost, white LEDs are still the most frequently used lighting source for most tissue culture labs and leafy green vegetable production. The research suggests that white LED’s might not be optimal for varied plant applications and some form of supplementation (specifically, the addition of R/FR) is beneficial for plant growth [3]. By adjusting and modulating lighting components, such as quantity (intensity) and quality (spectral composition), it is possible to extend harvest seasons and control plant morphology, such as plant size and shape, to optimize micropropagation [3].

In the visible spectrum, blue light is more energy-intensive and generally inhibits plant elongation while enhancing branch formation. Red light has the longest wavelength of visible light and promotes plant elongation, as well as leaf or bud growth, generating large amounts of biomass [4]. Far-red light (beyond 700 nm) has a low quantum yield (and is not considered in PAR) but can further alter plant morphology by increasing internode elongation and leaf expansion, which typically increases radiation capture in the shoot canopy, and thus yield [5]. Studies in lettuce have observed the effects of far-red light in leaf and canopy expansion, leading to a higher photosynthetic yield. This suggests that the addition of supplemental far-red has potential cost-saving opportunities for growers because far-red LEDs have higher efficiency [6].

In *Cannabis sativa*, light quality is one of the most crucial parameters, especially as operations are beginning to shift toward indoor, enclosed production systems [7]. For *cannabis* production, red light is chosen as it is associated with stem growth and rooting in other plants [7]. However, the relationship between red-to-blue ratios (either polychromatic or dichromatic) and the supplementation of far-red has been increasing for flower yield development and/or in vegetative cuttings. In micropropagation, and specifically in multiple-harvest systems, there is a need for elongated shoots, especially when planting ex vitro. Multiplication is usually promoted with plant growth regulators (PGR’s). A common issue with PGRs is hyperhydricity, where plantlets have a glassy appearance, nonfunctional stomata, and poor cuticles, and cannot control water loss, resulting in softened tissues and physical abnormalities [8]. Breaking apical dominance though repeated multiple harvests instead of using PGRs in agar-gelled medium allowed for three cycles of multiple harvests without hyperhydricity [9]. Using fed-batch liquid media overlays with an Oasis^®^ phenolic foam allowed for five cycles of repeated harvests without hyperhydricity [10], but shoots became shorter over repeated cutting cycles on both systems.

This study of light quality on shoot micropropagation tested seven lighting treatments of polychromatic white light and dichromatic red and blue light in a direct comparison, with and without far-red. The ratios were expressed as the percentage of blue light and the red-to-blue ratio. Observations were made of shoot production during five two-week cycles of repeated multiple harvests.

## 2. Results and Discussion

### 2.1. Number of Shoots Harvested

**Figure 1:**
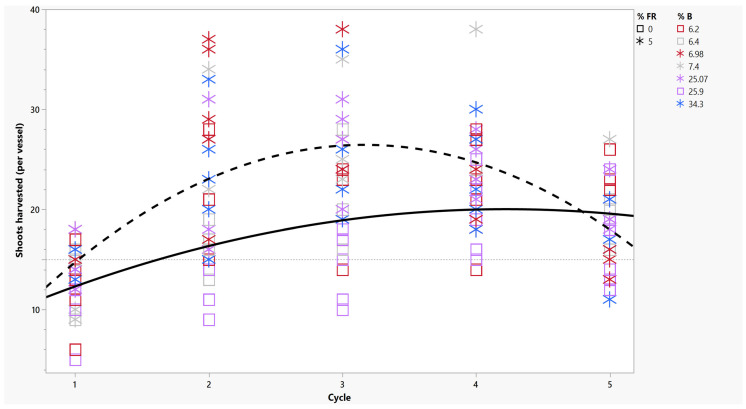
Number of shoots harvested per cycle from RV750 vessels with genotypes *Cannabis sativa* (‘Cherry 1’ and ‘BaOx’) with and without far-red-supplemented light and a varied percentage of blue light. Dashed line represents the regression of shoots harvested following far-red treatments per vessel; solid line represents the regression of treatments without far-red treatment. The initial 15 explants per vessel are indicated by the dotted reference line. Plants without far-red treatments are indicated by open boxes; far-red treatments are represented by asterisks, as indicated by the graph legend (% FR). Percentage of blue light is indicated by color, where gray is white light, red is high red, purple is medium red, and blue is low red, as indicated by the graph legend (%B).

Supplementing light treatments with 5% far-red light (Table 1) increased the number of shoots harvested over repeated cycles (Figure 1). During the first two-week growth cycle, an average plant under any far-red-supplemented treatment produced one elongated shoot (or 15 per vessel). Without far-red treatment, cycle 1 treatments were below the 1-to-1 production standards, producing approximately 10 shoots from a vessel of 15 explants; therefore, many shoots were not available for a repeated-cycle harvest two weeks later. Low-production vessels were typically obtained from treatments with a high percentage of blue (R_MID_) (Table 1).

Two weeks later, during the second harvest, the number of shoots harvested increased to 25 per vessel in plants under far-red light, while those without far-red treatment were still fewer than the original planting number of 15 (Figure 1). By cycle 3, 28 shoot tips were harvested from far-red treatments, while plants without far-red treatment were only producing 18 within the same harvest period (Figure 1). In *cannabis*, the in vitro hedging method was used to break apical dominance and promote lateral shoot regrowth without the use of exogenously applied cytokinin [9]. Since more shoots were harvested in cycle 1 from far-red treatments, lateral shoots quickly attained the desired length for recutting between the cycles. When an appropriate red/far-red treatment is applied, a high shoot multiplication rate is possible [11].

**Figure 2:**
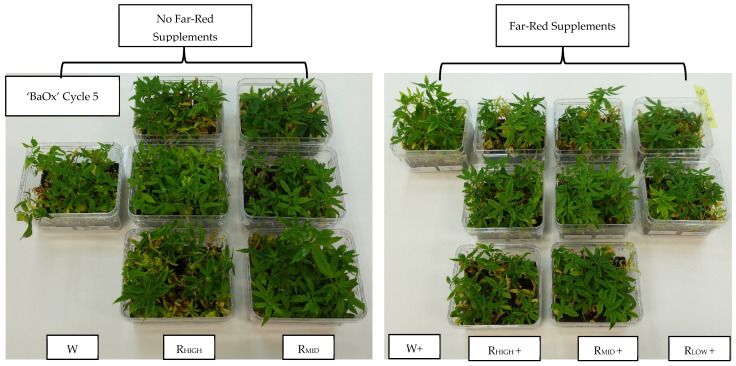
*Cannabis sativa* ‘BaOx’ planted in RV750 vessels at the end of five repeated-cycle harvests (10 weeks) based on polychromatic or dichromatic light source and supplementation of 5% far-red lighting.

After cycle 3, the number of shoots began to decline in both polychromatic and dichromatic light sources, even with the supplementation of 5% far-red (Figure 2). By cycle 5, the yield of harvested shoots returned to the original planting value of 15 per vessel with far-red and no far-red treatments (Figure 1). However, the total number of shoots produced in 10 weeks total was higher in plants grown under 5% far-red treatment regardless of the quality of PAR. Without far-red treatment, over five cycles, an approximate number of 84 shoots were produced, while with 5% far-red treatment, 109 shoots were produced from plants in vessels containing the original 15 initial explants. 

### 2.2. Length of Harvested Shoots

**Figure 3:**
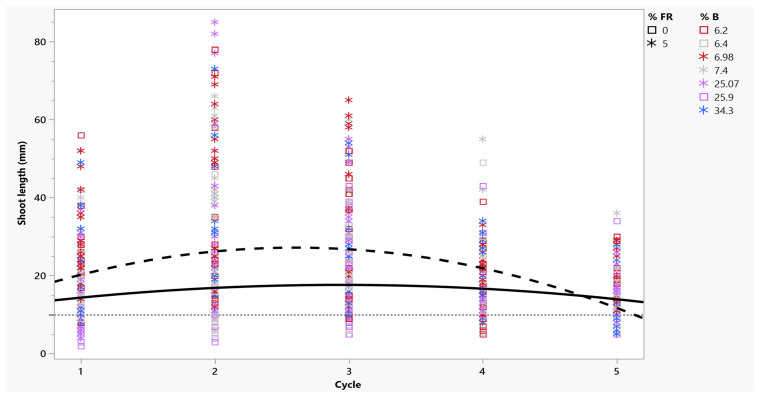
Length of shoots harvested per two-week cycle from RV750 vessels based on the percentage of blue light and far-red supplementation to two genotypes of *C. sativa* (‘Cherry 1’and ‘BaOx’). Dashed line represents regression of shoots harvested with far-red treatment per vessel; solid line represents regression of shoots harvested without far-red treatment. The desired harvest length of 10 mm is shown by the dotted reference line. Plants without far-red treatments are indicated by open boxes; far-red treatments are represented by asterisks, as indicated by the graph legend (% FR). The percentage of blue light is indicated by color, where gray is white light, red is high red, purple is medium red, and blue is low red, as indicated by the graph legend (% B).

**Figure 4:**
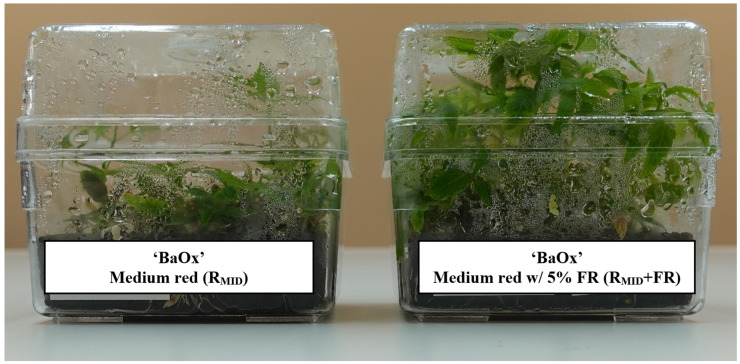
In vitro RV750 containing *Cannabis sativa* genotype ‘BaOx’ standard c during cycle 2 of repeated harvest after 4 weeks’ exposure to R_MID_ (left) and R_MID_ + FR (right) lighting treatments.

**Figure 5:**
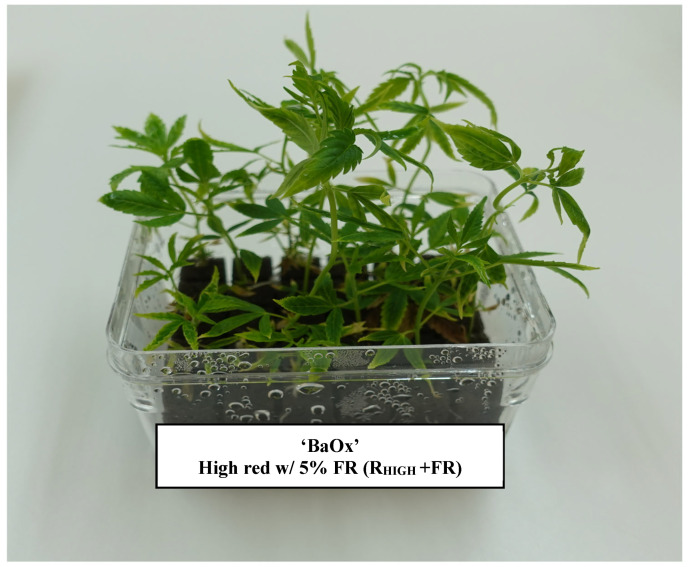
In vitro RV750 vessel containing *Cannabis sativa* genotype ‘BaOx’, displaying internode length variability before the first cycle harvest after 2-week exposure to R_HIGH_ + FR lighting treatment.

Not only were more shoots harvested with far-red, they were also more elongated, which aided in subsequent planting. The length of these shoots also increased over multiple cycles, and the increase was greater with the supplementation of far-red light to any polychromatic or dichromatic light source (Figure 3 and Figure 4). Starting in cycle 1, an average shoot exposed to 5% far-red treatment was 20 mm, and a shoot without far-red treatment was 14 mm (Figure 3). Regardless of far-red treatment, micro-cuttings were an adequate planting size for mist bed rooting (>10 mm). In far-red treatments, specifically with high red-to-blue ratios, (W + FR, R_HIGH_ + FR) large variations in shoot length occurred, where some shoots had a single internode of 80 mm that touched the top of the vessel, while others were 20 mm (Figure 5). This variation in shoot length might not be acceptable to commercial growers, as they are looking for genetically uniform, identical plants [12].

Blue light had a significant (Table 2) effect on shoot length, as plants with a lower R:B ratio (high %B) were often shorter. In greenhouse studies of *cannabis*, LED treatments with high blue fractions similarly reduced plant heights. This was expected as a high percentage of blue light is known to decrease growth, such as leaf expansion, stem elongation, and overall growth [13]. In the first cycle, without far-red treatment, 31 of the 77 shoots harvested did not meet the desired 10 mm planting standard. When supplemented with far-red treatment, only 22 of the 116 plants harvested failed due to their similarly high ratios of blue.

Over the next repeated cycles of harvest, shoot length increased and was greater in far-red treatments (Figure 4). In treatments with the lowest percentage of blue light (including white), plants touched the tops of the vessels (up to 60 mm) by cycle 3 with far-red treatment (Figure 3). An average shoot length during this cycle was 26 mm from plants treated with far-red and 18 mm for those that were not (Figure 3). After cycle 3, the shoot length of plants decreased in both far-red treatments, regardless of LED source, as the number of shoots also declined. By cycle 5, non-far-red-treated plants were 12 mm, shorter than plants with far-red treatment, which were 15 mm (Figure 2 and Figure 3). Overall, over five cycles, 65% of harvested shoots grown without far-red treatment were below the standard of 10 mm, while only 10% were below that standard when treated with far-red supplements.

### 2.3. Dry Shoot Mass

**Figure 6:**
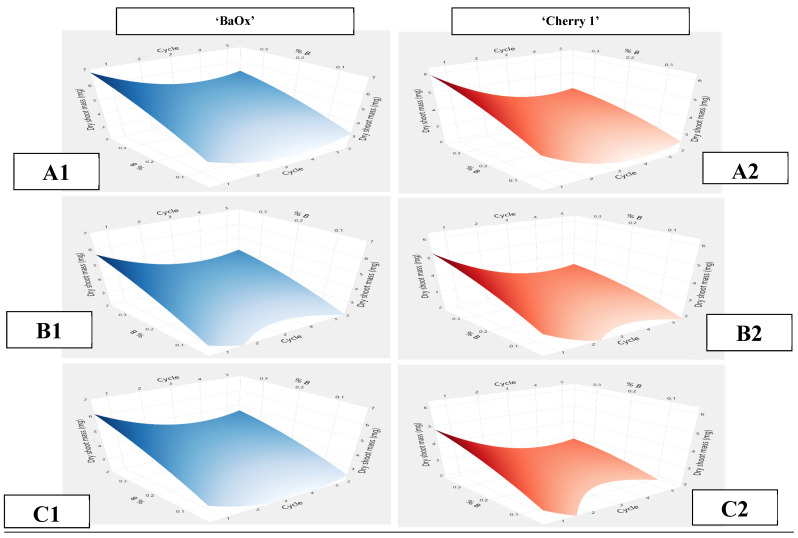
Dry mass per shoot harvested from RV750 vessel over five harvest cycles for *Cannabis sativa* genotypes ‘BaOx’, indicated by blue (**left**), and ‘Cherry 1’, indicated in red (**right**), based on the percentage of blue light in polychromatic and dichromatic treatments. Initial planting standards of tissue were designated as ‘BaOx’ (**A1**–**C1**) and ‘Cherry 1’ (**A2**–**C2**).

Far-red light supplementation to any polychromatic or dichromatic light treatment increased shoot production and length but had no effect on dry mass or ex vitro survival. Over multiple harvest cycles, dry shoot mass was most influenced by the percentage of blue light, and there was a significant genotypic difference (Table 2). Regardless of far-red treatment, the higher percentage of blue light in dichromatic or polychromatic R:B ratios led to an increase in dry mass over multiple cycles. The initial sorting by shoot standard was also significant, as “a” standard plant always had the highest proportion of dry mass, and the “c” standard had the lowest, when defined as the proportion of initial mass at planting. Genotype differences also occurred, as ‘BaOx’ had an overall higher dry mass for all cycles and standard ratings. In cycle 1, ‘BaOx’ (“a”) plants, had an average dry shoot mass of 7 mg when exposed to a high percentage of blue light (R_MID_). Under the lowest percentage of blue light (W, R_HIGH_), dry shoot mass was only 2 mg (Figure 6). Dry shoot mass decreased between cycles 2 and 3, where it remained constant at around 4 mg (Figure 6). The smaller initial explant tissues (b and c) followed the same trend, decreasing with continuous cycles. In the genotype ‘Cherry 1’, dry shoot mass during cycle 1 was 6 mg with high blue light (R_MID_), but under R_HIGH_ and W, dry mass was only 2 mg (Figure 3). Similarly, ‘BaOx’ dry mass decreased between cycles 2 and 3 and remained constant during the remaining cycles, at approximately 4 mg. Standards “b” and “c” decreased similarly, with cycle 1 being 5 mg and remaining constant at 3 mg at the end of cycle 3. By decreasing the standards from “a” to “c”, dry mass decreased by 18% in ‘Cherry 1’ and 22% in ‘BaOx’ over multiple cycles. Decreasing from “b” to “c” decreased dry mass by 8% in ‘BaOx’ and 16% in ‘Cherry 1’.

### 2.4. Ex Vitro Rooting

Regardless of in vitro treatments prior to ex vitro rooting, there were no significant effects reported on greenhouse growth. Sixty eight percent of plants were rooted on the greenhouse mist bench. The highest percentage of rooting (80%) occurred during cycles one and five, although these shoots were arguably not of the best visual quality. During the intermediate cycles, rooting was 60% and coincided with a record-high outdoor temperature of 26 °C; normal averages are usually 13 °C in November. The warmer temperatures coincided with a period of poor rooting success. Plants received hand irrigation during dry periods, but the first 24 h ex vitro are a critical period for dry-down and far-red-treated plants wilted excessively due to their size. An increase in shoot elongation under far-red LED’s was ascribed to stem fragility because of the excessive elongation of the internode [2]. Stress during acclimatization from the in vitro to the ex vitro conditions is especially acute during the rooting of micro-shoots. Desiccation is the largest cause of failure. Rooting acclimatization requires the gradual adaptation of in vitro lab plants to the greenhouse environment, where leaves maintain water levels with an increased vapor pressure deficit and irradiance while the new root system establishes its adequate water uptake.

## 3. Methods and Materials

Fifteen apical nodes or shoot tips of approximately 1–2 cm in length, belonging to *C. sativa* (‘Cherry 1’and ‘BaOx’), were planted in RV750 (Smithers Oasis Kent, OH) vessels with vented lids containing 120 mL of DKW medium (OIL) (Driver and Kuniyuki, Davis, CA, USA 1984) and supplemented with 3% *w*/*v* sucrose, pH 6.2, and Oasis^®^ IVE (Smithers—Oasis Company, Kent, OH, USA). Vessels were planted based on initial shoot standard, where “a” represented a standard vessel contained the visually largest shoot tissues; thus, “b” and “c” were smaller. After planting, vessels were sealed with PVC sealing film (PhytoTech Labs, Lenexa, KS, USA), before being placed into seven lighting treatments, with each treatment containing one “a”, “b”, and “c” vessel per genotype.

The lighting treatments shown in Table 2 were applied as LEDs (OSRAM Phytofy^®^ RL, Wilmington, MA, USA) for a 16-hour photoperiod with a similar PPFD (190–240 µmol m^−2^ s^−1^). The uniformity of the spectral distribution and the light intensity of each chamber were verified using a calibrated spectroradiometer LI-190R (LICOR Biosciences, LiCor, Inc., Lincoln, NE, USA).

After two weeks, apical shoot tips were harvested and counted, and fresh mass was determined. The length of five randomly chosen shoot tips was measured (base of cut stem to apical shoot tip) and the number of leaves was recorded. The same five shoots were analyzed for dry mass after being enclosed in paper envelopes for 72 h in a convection-drying oven (Thermo Fisher Scientific, Pittsburgh, PA, USA) at 60 °C. An aseptic supplement of 15 mL DKW was added to each vessel, and all were rewrapped with PVC sealing film (PhytoTech Labs, Lenexa, KS, USA) until the next harvest.

Five random shoot tips were also placed in Petri dishes and planted ex vitro in the greenhouse under mist on the 4th and 18th October, and 1st, 15th, and 29th November 2023 at Global Positioning System (GPS) coordinates 34.67, −82.83. Cuttings were planted in single-cell hexagon standard vacuum plug trays (The HC Companies, Twinsburg, OH, USA) that were filled with Fafard 3B soilless medium (Sungro Horticulture, Agawam, MA, USA). Prior to planting, explants received a quick dip in 1000 ppm K-IBA. For the first 24 h, humidity domes (T.O. Plastics, Clearwater, MN, USA) were placed over the trays in increment periods depending on the weather conditions and were irrigated via hand misting when necessary. After 16 days, cuttings were measured for shoot length, number of leaves, and rooting (plants that firmly held a root ball when removed from the cell were given a 1, while anything less was given a 0). Rooted plants were up potted into 2-gallon pots (Poppelmann Plastics USA, Claremont, NC, USA), with up to four per pot, and were 3 inches apart; then, they received 150 ppm N of Peters Professional 20-20-20 (N-P_2_O_5_-K_2_O) (Allentown, PA, USA) fertigation on alternate days for six weeks. At this time, plants were analyzed to determine their height, longest leaf, and stem–leaf ratios to determine apical or multiple branching patterns. This process was repeated for five cycles, with extra irrigation applied during cycles 2–3 due to the extremely high fall temperatures. Data were analyzed using JMP 16.1 (SAS Inst., Cary, NC, USA) with factors considered significant using a *p*-value of 0.001.

## 4. Conclusions

Supplementation with 5% far-red lighting increased the shoot number and shoot length of in vitro plants under any tested dichromatic or polychromatic light treatment. Through multiple harvests, a higher total of accumulated high-quality shoots was produced with far-red supplements. Through multiple harvests, both genotypes responded similarly to the effects of far-red light. The percentage of blue light did not increase the yield of shoots over multiple cycles in vitro, but did increase the dry mass of shoots, although there was no significant correlation between dry mass and ex vitro growth.

White LEDs are often installed in tissue culture labs, and can come in both cool (more blue) and warm versions (more red) for the consumer market, making it possible to select them based on different plant morphological needs. As this work suggests, far-red supplementation would benefit LEDs with an optimal R:B ratio 3:1 (R_MID_ + FR), obtaining uniform shoots that are high in dry mass for five repeated cycles. There was also no correlation between far-red light and ex vitro growth.

The breaking of apical dominance was accomplished through multiple cuttings of the apical meristem at harvest using the hedging system, rather than exogenous cytokinin applications, which helps avoid issues that often hinder *cannabis* tissue culture, such as hyperhydricity. Through nutrient supplementation in the modified hedging and fed-batch system using Oasis^®^ IVE and light-signaling responses with far-red light, multiplication rates were increased with a shorter interval time (two weeks) between harvests. An ideal lighting solution would allow uniform plants to be cut on a regularly scheduled harvest period for ex vitro transfer. Future work could examine the duration, intensity, and frequency of far-red lighting on shoot quality. Further improvements in the ex vitro rooting environment would be complementary to the anticipated improvement in shoot quality.

## Figures and Tables

**Table 1:** Overview of dichromatic (R:B) and polychromatic (white) light treatments used, along with their red-to-blue ratio and percentage of blue (B) calculated using total measured PPFD (µmol m^−2^ s^−1^).

Light Treatment	R:B	B (%)	Measured Total PPFD. (µmol m^−2^ s^−1^)
White (W)	9.1	6.4	211
High red (R_HIGH_)	15.2	6.2	209
Medium red (R_MID_)	2.9	25.9	238
White w/5% far-red (W + FR)	7.8	7.4	192
High red w/5% far-red (R_HIGH_ + FR)	13.3	6.9	214
Medium red w/5% far-red (R_MID_ + FR)	3.0	25.1	243
Low red w/5% far-red (R_LOW_ + FR)	1.9	34.3	198

**Table 2:** Summary ANOVA of shoots harvested, shoot length, dry shoot mass, and percentage of rooted explant survival for genotypes ‘Cherry 1’and ‘BaOx’ in response to source factors cycle (C), genotype (G), far-red (FR), % blue (B), and standard (S) over five repeated cycle harvest cuttings.

Source	Shoots Harvested	Shoot Length	Dry Mass per Shoot	Ex Vitro Rooting
Cycle (C)	<0.0001	0.0597	<0.0001	0.2724
C × C	<0.0001	<0.0001	0.0040	0.0022
Genotype (G)	0.7085	0.7892	0.0008	0.1578
Far-red (FR)	<0.0001	<0.0001	0.0679	0.6218
% Blue (B)	0.0025	<0.0001	<0.0001	0.3943
Standard (S)	0.4636	0.0051	<0.0001	0.0787
C × G	0.0505	0.3263	0.4218	0.3172
C × FR	0.1238	0.0001	0.1750	0.5680
C × B	0.9931	0.0744	0.4135	0.8669
G × FR	0.9145	0.2100	0.5090	0.0102
G × B	0.2010	0.1135	0.1830	0.5226
FR × B	0.1207	0.8704	0.0937	0.6359
B × B	0.9986	0.5571	0.6067	0.3307
Whole model $R^{2}$	0.4550	0.1718	0.4631	0.0400

## Data Availability

The raw data supporting the conclusions of this article will be made available by the corresponding authors on request.
